# Artificial intelligence-assisted optimization of extraction process, characterization, and functional analysis of globulin from safflower seed meal

**DOI:** 10.3389/fnut.2025.1708593

**Published:** 2025-11-17

**Authors:** Keer Xiao, Qiaoyu Wang, Xinyu Meng, Ziteng Zhao, Mukaddas Sai, Lili Guan, Qiuyu Lu, Lingyu Gao, Jing Yang, Linna Du

**Affiliations:** 1 Engineering Research Center of the Chinese Ministry of Education for Bioreactor and Pharmaceutical Development, College of Life Science, Jilin Agricultural University, Changchun, China; 2 Institute for Safflower Industry Research of Shihezi University/Pharmacy College of Shihezi University/Key Laboratory of Xinjiang Phytomedicine Resource and Utilization, Ministry of Education, Shihezi, China

**Keywords:** safflower, seed meal, globulin, extraction, functional properties

## Abstract

Safflower seed meal, a protein-rich byproduct of oil extraction, is often discarded as waste, resulting in both resource inefficiency and environmental concerns. In this study, albumin, globulin, prolamin, and glutelin were sequentially extracted from safflower seed meal via Osborne extraction method, accounting for 22.69%, 27.69%, 37.33%, and 12.28% of the total protein in the meal, respectively. Physicochemical and functional characterizations revealed distinct functional advantages among the protein fractions. Specifically, the globulin fraction demonstrated high foaming property, favorable emulsifying capacity, and strong surface hydrophobicity, whereas albumin and glutelin exhibited good oil-holding capacity and water-holding capacity, respectively. Given globulin's outstanding performance, its extraction process was further optimized using artificial intelligence-assisted approaches. The suitable extraction conditions for globulin were determined as follows: extraction time of 110 min, solid-liquid ratio of 1:47 g/mL, extraction temperature of 37 °C, and NaCl concentration of 1.24 mol/L. Under these conditions, the globulin yield reached 7.33 ± 0.10%. SDS-PAGE analysis indicated that the molecular weight of globulin was characterized by small molecular weights (13–53 kDa). FTIR spectra revealed β-sheet (30%) was the dominant secondary structure of globulin, while the α-helix content was the lowest (18%); this structural feature may contribute to the globulin's high foaming and emulsifying capabilities. Amino acid analysis identified 17 amino acids in globulin, including eight essential amino acids, with hydrophobic amino acids accounting for 46.88%. Collectively, these results demonstrate that safflower seed meal-derived globulin is a nutritionally balanced and functionally potent plant protein, exhibiting great application potential in the food field.

## 1 Introduction

With the growth of the world's population, animal-derived proteins have been unable to meet the increasing demand for protein, while plant-derived proteins have received rising interest due to their abundant availability, rapid renewability, and cost-effectiveness (1, 2). Moreover, their nutritional and functional variability has further drawn considerable focus to industrial applications of these proteins (3). Importantly, compared with animal protein intake, regular consumption of plant proteins offers greater benefits in mitigating the health and environmental risks associated with excessive animal protein consumption, such as hyperlipidemia, hypertension, and diabetes (4). Beyond direct utilization as food ingredients, plant proteins are also widely applied in the food additives or as raw materials for packaging films (5). Thus, plant proteins are gradually recognized as a promising option in the food (6), pharmaceutical (7), and cosmetics (8) industries. Despite their promising prospects, commercially available plant-based proteins currently rely on a narrow range of sources, such as soybeans, peas, rapeseed, and corn (9). With the fast expansion of the plant protein consumer market, this limited source range has led to a supply shortage, thereby highlighting an urgent need to diversify plant protein sources.

Safflower (*Carthamus tinctorius* L.), a traditional oilseed crop belonging to the Asteraceae family, is extensively cultivated worldwide (10). Beyond its historical use as a dye source, accumulating evidence highlights the potential of safflower in medicine, cosmetics, and oil production (11). Currently, the diverse health benefits of this crop have been well documented (12, 13). Moreover, safflower seeds are rich in unsaturated fatty acids, particularly linolenic acid, which exhibit extensive biological activities, including immune regulation (14), anti-obesity effects (15), anti-neuroinflammation properties (16). Additionally, safflower oil has been found to possess longer shelf life and stronger oxidative stability; these attributes enhance its suitability for deep frying applications while maintaining health benefits (17). Therefore, safflower seeds are widely recognized as a critical source of high-quality edible oil.

Owing to the considerable multifunctional values of safflower oil, safflower is extensively cultivated in over 20 countries worldwide, supporting a global safflower seed production of up to 6.3 million tons annually (17). However, in contrast to the widespread utilization of safflower oil, the large quantity of seed meal, a byproduct of oil extraction, is often neglected and has not been effectively exploited in a comprehensive manner (18). Conventionally, safflower seed meal is only directly added to animal feed, which results in inefficient utilization of this valuable resource. Phytochemical investigations have identified multiple bioactive and nutritional substances in safflower seed meal, including proteins, flavonoids, lignans, fibers (19, 20), with protein content being particularly prominent at over 40% (21). Furthermore, the digestibility of proteins isolated from safflower seed meal has been reported to be as high as 78% (22). Collectively, these findings indicate that safflower seed meal is a low-cost and high-value protein source, thereby highlighting the particular importance of exploring its broader application potential.

Empirically, plant seed proteins are typically categorized into four distinct fractions based on their solubility, namely globulin (saline-soluble proteins), albumin (water-soluble proteins), prolamin (alcohol-soluble proteins), and glutelin (alkali-soluble proteins). These fractions are commonly isolated via Osborne extraction method, which is favored by its simplicity and ease of operation (4, 23). As widely recognized, the physicochemical and functional properties of these proteins are critical for determining their industrial applicability. However, plant proteins are highly susceptible to structural changes induced by various factors (sample source, extraction condition, etc.), which in turn alter the proteins' functional performance and biological behavior (4). Therefore, optimizing the extraction parameters of safflower protein and systematically analyzing its physicochemical and functional properties are of great significance for promoting its industrial utilization.

In the present study, four protein fractions (albumin, globulin, prolamin, and glutelin) were separated from safflower seed meal via the Osborne extraction method, and their physicochemical and functional properties were firstly investigated. Subsequently, response surface methodology (RSM) and artificial neural network-genetic algorithm (ANN-GA) were employed to further optimize the conditions for globulin extraction. Finally, additional physicochemical properties of globulin derived from safflower seed meal were explored, including molecular weight, amino acid composition, and Fourier transform infrared spectroscopic characteristics. This study not only provides a novel approach for the value-added utilization of safflower seed meal, but also highlights the potential of the extracted globulin as a promising plant protein ingredient for food formulations.

## 2 Materials and methods

### 2.1 Materials

Safflower seed meal was provided by Xinjiang Jincheng Hengda Trading Co., Ltd. (Tacheng, China) and ground into fine powder (60 mesh) using a high-speed crusher (YB-4500A, YUNBANG, China). Safflower seed meal was then defatted twice using n-hexane at a solid-liquid ratio of 1:3 (g/mL) for 1.5 h at room temperature. The residual solvent was removed by rotary evaporation (24). Subsequently, the defatted seed meal powder was freeze-dried using a Genesis2000SQ lyophilizer (VIRTIS, USA) and further pulverized to pass through a 60-mesh sieve. ANS (8-anilino-1-naphthalenesulfonic acid) were supplied by Shanghai Yuanye Biotechnology Co., Ltd. (Shanghai, China). Sodium hydroxide and hydrochloric acid were obtained from Laiyang Kangde Chemical Co. Ltd. (Yantai, China). Other analytical grade chemicals were purchased from Shanghai Yuanye Biotechnology Co., Ltd.

### 2.2 Proximate analysis of safflower seed meal

The proximate composition of safflower seed meal was detected in line with previous studies (23, 25), including moisture, ash, fat, crude protein, carbohydrate, and cellulose.

### 2.3 Preparation of globulin, albumin, prolamin, glutelin from safflower seed meal

Sequential Osborne extraction was employed to isolate proteins from the safflower seed meal (26). Briefly, 20 g of the defatted meal powder was mixed thoroughly with 200 mL deionized water, and the mixture was magnetically stirred at 1,000 rpm for 100 min at 25 °C to extract proteins. After centrifugation (8,000 rpm, 25 min), the supernatant 1 containing albumin and precipitate 1 were harvested separately. Subsequently, 1 mol/L NaCl solution was added to the precipitate 1 at a solid-liquid ratio of 1:10 (g/mL), and the mixture was agitated for another 100 min (1,000 rpm, 25 °C). To obtain the globulin extract, centrifugation was conducted at 8,000 rpm. Twenty-five minutes later, the supernatant 2 was obtained, which was the globulin extract of safflower seed meal. The remaining precipitate 2 was retained for prolamin and glutelin extraction. For prolamin extraction, the precipitate 2 was mixed thoroughly with 70% ethanol at a solid-liquid ratio of 1:10 g/mL, and extraction was conducted at 25 °C for 100 min (1,000 rpm). Similarly, the supernatant 3 and precipitate 3 were harvested by centrifugation with the same parameters as the above steps, and the supernatant 3 was prolamin. Finally, the residue 3 was mixed with alkaline solution (pH 7, adjusted with 1 mol/L NaOH), followed by sequential glutelin extraction and centrifugation (parameters as above). The resulting supernatant 4 was collected as the glutelin extract.

### 2.4 Determination of protein content and recovery efficiency

BCA kit purchased from Solarbio (PC002, Beijing, China) was introduced to detect the protein concentration of four protein fractions. The recovery rates of protein fractions were calculated in line with a previous study (23) using the following formula:


$$\begin{array}{l} Recovery\;rate\;\left(\%\right)\;=\frac{P}{S}\;\times \;100 \end{array}$$ (1)

where *P* represents the weight of the protein fractions (g), and *S* denotes the weight of the defatted safflower seed meal powder employed for protein extraction (g).

### 2.5 Solubility analysis of protein fractions prepared from safflower seed meal

Each tested protein fraction (0.1 g) was dispersed in 10 mL deionized water individually. The pH of each protein dispersion was then adjusted to 3.0, 5.0, 7.0, 9.0, and 11.0 using NaOH (0.5 mol/L) and HCl (0.5 mol/L) solutions. After centrifuging the resulting dispersions at 12,000 g for 30 min at 25 °C, the protein content in the supernatant was determined using the BCA kit (27).

### 2.6 Surface hydrophobicity detection

Surface hydrophobicity (*S*_o_) of protein fraction was measured following the method described by Qin et al. (28), using ANS as the fluorescent probe. Briefly, the protein samples were serially diluted with 0.01 M Tris-HCl buffer (pH 7.0) to final concentrations of 0.08, 0.16, 0.24, 0.32, and 0.40 mg/mL. Subsequently, 3 mL of each diluted sample was mixed with 50 μL of 8.0 mmol/L ANS solution and incubated in the dark at room temperature for 20 min. Fluorescence measurements were then performed using an FP-6500 spectrofluorometer (Jasco, Japan). The excitation wavelength, emission wavelength, and emission slit width were set at 390 nm, 400–550 nm, and 5 nm, respectively.

### 2.7 Analysis of the functional properties of protein fractions from safflower seed meal

In line with the method outlined by Pearce et al. (29), emulsifying capacity (EC) of samples were determined. Briefly, 6 mg protein samples were thoroughly mixed with 6 mL distilled water, after which 2 mL corn oil was added. The mixture was homogenized using a homogenizer (HR-500D, HUXI Corporation, Shanghai, China) at 20,000 rpm for 60 s. Following homogenization, a 25 μL aliquot was diluted 100-fold in 0.1% (*w*/*v*) sodium dodecyl sulfate solution (SDS). Following a 10-min equilibration, the absorbance of the emulsion was measured at 500 nm using a UV-Vis spectrophotometer (754N, INESA, China). Subsequently, EC was evaluated by determining the emulsifying activity index (EAI) and emulsifying stability index (ESI), respectively, following the method described by Li et al. (30). The calculations of EAI and ESI were performed using the following equations:


$$\begin{array}{l} EAI\;\left(m^{2}/g\right)\;=\frac{\;2\times 2.303\times A_{0}\times D}{c\times \phi \times \theta \times 10,000} \end{array}$$ (2)

$$\begin{array}{l} ESI\;\left(\min\right)\;=\;\frac{A_{0}\times \Delta t}{A_{0}-A_{10}} \end{array}$$ (3)

In these equations, *c, D*, θ, ϕ, and Δ*t* represent the protein concentration, dilution multiple, optical path (cm), oil volume fraction in emulsion and a time interval of 10 min, respectively. *A*_0_ and *A*_10_ are the emulsion absorbance at 0 and 10 min.

For foaming properties, protein samples suspended in distilled water (10 mg/mL) were homogenized using a homogenizer at 10,000 rpm for 2 min. The foam volume was determined immediately after homogenization and again after a 10 min interval. The foaming capacity (FC) and foaming stability (FS) were subsequently calculated using Equations 4, 5, respectively (31).


$$\begin{array}{l} FC\;\left(\%\right)\;=\;\frac{V_{0}-V_{i}}{V_{i}}\;\times \;100 \end{array}$$ (4)

$$\begin{array}{l} FS\;\left(\%\right)\;=\;\frac{V_{10}-V_{i}}{V_{i}}\;\times \;100 \end{array}$$ (5)

where *V*_*i*_ is the volume of the solution before mixing, *V*_0_ is the volume of the solution after mixing at 0 min and *V*_10_ is the volume of the solution after mixing at 10 min.

Additionally, the water-holding capacity (WHC) and oil-holding capacity (OHC) of the protein isolates were measured (32). In brief, 0.01 g of dried protein sample (denoted as M_0_) was dispersed in a pre-weighed centrifuge tube (1.5 mL, initial weight: M_1_) containing 1 mL of deionized water or soybean oil. The dispersion was vortexed for 30 s and subsequently centrifuged at 5,000 rpm for 15 min. After decanting the supernatant, the tube containing the protein residue (M_2_) was weighed again. The WHC and OHC (g/g) were then calculated using the following formula:


$$\begin{array}{l} WHC/OHC\;\left(g/g\right)\;=\;\frac{M_{2}-M_{1}-M_{0}}{M_{0}} \end{array}$$ (6)

### 2.8 Optimization of extraction process for globulin from safflower seed meal

To enhance the extraction efficiency of globulin from safflower seed meal, the extraction parameters were optimized using single-factor test and Box-Behnken design (BBD) experiment.

#### 2.8.1 Single-factor test

To evaluate the impacts of different parameters on globulin extraction efficiency, single-factor tests were introduced. Firstly, following the methods described in Section 2.3, the globulin was extracted from precipitate 1 for 100, 105, 110, 115, and 120 min, respectively. The solid-liquid ratio, extraction temperature, and NaCl concentration were fixed at 1:20 g/mL, 25 °C, and 1 mol/L, respectively. Then, based on extraction efficiency, the time point yielding the highest globulin content was selected for subsequent experiments. Secondly, to assess the impact of solid-liquid ratio on the target response value, 10 g of precipitate 1 were added to 1 mol/L NaCl solutions with volumes of 200, 250, 300, 350 and 400 mL, respectively. After well mixing, the mixture was stirred at 25 °C for 110 min. Similarly, the globulin extraction yield was calculated as the response variable. Thirdly, globulin was extracted using NaCl solutions of varying concentrations (0.5, 0.75, 1, 1.25, 1.5 mol/L) for 110 min at 25 °C, and the mass of volume ratio between precipitate 1 and NaCl solutions was set to 1:35 g/mL. Finally, the temperature suitable for globulin extraction was screened in five temperature ranges of 25, 30, 35, 40, and 45 °C, while maintaining other parameters at their optimal levels. All above experiments were performed in triplicate.

#### 2.8.2 Box-Behnken design experiment

To further determine the optimal value of the four factors, a BBD consisting of 29 experiments (including five center point replicates) was used. The design matrix of BBD is presented in Table 1. Following the experimental scheme outlined in the table, batch extractions of globulin from safflower seed meal were performed under varying conditions, and the corresponding globulin yields were determined. Furthermore, two approaches were employed to analyze the resulting data, including response surface methodology (RSM) and artificial neural networks coupled with genetic algorithms (ANN-GA).

**Table 1:** The design matrix and results of Box-Behnken design experiment.

**Run**	***A*: Extraction time (min)**	***B*: Solid-liquid ratio (g/mL)**	***C*: Extraction temperature (°C)**	***D*: NaCl concentration (mol/L)**	**Extraction yield of globulin (%)**
1	0 (110)	0 (1:35)	1 (50)	1 (1.5)	6.26 ± 0.04
2	1 (120)	0 (1:35)	1 (50)	0 (1)	6.08 ± 0.04
3	0 (110)	0 (1:35)	0 (40)	0 (1)	5.66 ± 0.05
4	0 (110)	1 (1:50)	−1 (30)	0 (1)	6.28 ± 0.12
5	0 (110)	0 (1:35)	0 (40)	1 (1.5)	5.45 ± 0.10
6	0 (110)	1 (1:50)	1 (50)	0 (1)	6.15 ± 0.12
7	0 (110)	0 (1:35)	0 (40)	0 (1)	6.47 ± 0.05
8	0 (110)	0 (1:35)	0 (40)	0 (1)	6.35 ± 0.14
9	1 (120)	−1 (1:20)	0 (40)	0 (1)	2.96 ± 0.06
10	0 (110)	−1 (1:20)	0 (40)	1 (1.5)	4.29 ± 0.02
11	−1 (100)	0 (1:35)	0 (40)	−1 (0.5)	4.85 ± 0.08
12	0 (110)	−1 (1:20)	0 (40)	−1 (0.5)	3.26 ± 0.02
13	0 (110)	0 (1:35)	−1 (30)	−1 (0.5)	4.80 ± 0.09
14	1 (120)	0 (1:35)	0 (40)	−1 (0.5)	4.82 ± 0.04
15	0 (110)	0 (1:35)	0 (40)	0 (1)	6.28 ± 0.02
16	−1 (100)	0 (1:35)	−1 (30)	0 (1)	5.26 ± 0.04
17	0 (110)	0 (1:35)	1 (50)	−1 (0.5)	5.42 ± 0.06
18	0 (110)	1 (1:50)	0 (40)	−1 (0.5)	5.23 ± 0.11
19	1 (120)	0 (1:35)	0 (40)	1 (1.5)	5.42 ± 0.03
20	0 (110)	−1 (1:20)	−1 (30)	0 (1)	3.80 ± 0.03
21	1 (120)	1 (1:50)	0 (40)	0 (1)	6.80 ± 0.07
22	−1 (100)	0 (1:35)	1 (50)	0 (1)	6.79 ± 0.10
23	0 (110)	0 (1:35)	0 (40)	0 (1)	6.38 ± 0.17
24	0 (110)	0 (1:35)	−1 (30)	1 (1.5)	5.38 ± 0.03
25	0 (110)	−1 (1:20)	1 (50)	0 (1)	4.34 ± 0.05
26	0 (110)	1 (1:50)	0 (40)	1 (1.5)	6.46 ± 0.07
27	−1 (100)	−1 (1:20)	0 (40)	0 (1)	4.35 ± 0.02
28	−1 (100)	1 (1:50)	0 (40)	0 (1)	6.49 ± 0.10
29	1 (120)	0 (1:35)	−1 (30)	0 (1)	6.50 ± 0.03

For RSM, a multiple quadratic regression (MQR) equation was established by Design-Expert13 software to represent the relationship between the response value and four optimization factors. To evaluate the model adequacy and the statistical significance of each factor, analysis of variance (ANOVA) was performed using the same software. Several diagnostic plots (perturbation plot, normal probability distribution, plots of predicted and actual values, and diagram of residuals vs. number of runs) were drawn using Design-Expert13 software. Furthermore, response surface plots and contour plots were constructed to visually elucidate the interactive effects between factors.

For ANN-GA analysis, a three-layer ANN model was used to fit the data using MATLAB software (R2006a, Math-Works Inc., Natick, MA, USA). Among the 29 experimental sets in the BBD, 21, 4, and 4 sets were selected for training, testing and verification, respectively. The Levenberg-Marquardt method was employed for model training. The number of epochs, learning rate, and activation function were 1,000, 0.01, and sigmoid, respectively. In accordance with other research, the degree of approximation (*D*a) values were calculated for different numbers of hidden layer nodes to optimize the network architecture (33). In this formula, the constant was set to 12. Subsequently, the GA optimization process was implemented referring to the method highlighted in the literature (34), with the parameters configured as follows: population type = double vector, selection function = stochastic uniform, population size = 20, crossover fraction = 0.8, the initial population = given randomly, elite count = 2, crossover function = scattered, migration fraction = 0.2, penalty factor = 100, and migration interval = 20.

#### 2.8.3 Verification experiments

To verify the reliability of the optimized conditions, five independent replicates of globulin extraction were conducted under the optimal conditions predicted by MQR and ANN models, respectively. Subsequently, the error between the predicted maximum globulin extraction yield and the actual extraction yield from the two models were further calculated to quantify the predictive accuracy of each model. Additionally, in line with previous studies (35, 36), root mean square error (RMSE), mean square error (MSE), coefficient of determination (*R*^2^), absolute average relative deviation (AARD), and average relative error (ARE) of these two models were calculated, respectively.

### 2.9 Sodium dodecyl sulfate-polyacrylamide gel electrophoresis (SDS-PAGE)

The molecular weight of globulin was analyzed by reducing and non-reducing SDS-PAGE following the protocol described by Xiao et al. with minor modifications (37). Briefly, globulin extracts (2 mg/mL) were mixed with loading buffer at a ratio of 1:1 (v/v) in the presence or absence of β-mercaptoethanol (1:25, v/v). The mixtures were maintained at 100 °C for 5 min, and 10 μL of the mixture were then subjected to SDS-PAGE as other work.

### 2.10 Fourier transform infrared spectroscopy (FTIR)

FTIR spectra of globulin extract (550–4,000 cm^−1^) was recorded using a Nicolet 380 spectrometer (Thermo Scientific, UK). All spectra were subsequently corrected using EZ Omnic (v7.3). For the analysis of protein secondary structures, the amide I band (1,600–1,700 cm^−1^) in the FTIR spectra was deconvoluted via Gaussian fitting combined with second-derivative analysis (PeakFit v4.12). The relative content of each secondary structure was then calculated based on the integrated area of the deconvoluted peaks corresponding to specific secondary structure components.

### 2.11 Determination of amino acid composition

Globulin extracted from safflower seed meal was accurately weighed and transferred into an anaerobic hydrolysis tube. Subsequently, 5 mL of HCl (6 mol/L) was added to the tube, and the mixture was flash-frozen using liquid nitrogen. After complete solidification, the tube was vacuum-sealed and hydrolyzed at 110 °C for 13 h in a constant-temperature drying oven. Following cooling to room temperature, the hydrolysate was diluted to a final volume of 10 mL and filtered through a 0.45 μm aqueous filter membrane to remove particulate impurities. A 0.5 mL aliquot of the filtrate was transferred into an Eppendorf tube and dried under vacuum. The resulting residue was reconstituted in 1 mL of deionized water and dried again; this step was repeated twice. Finally, the residue was dissolved in 1 mL of pH 2.2 sample dilution buffer and filtered through a 0.22 μm aqueous filter membrane. The amino acid composition of the globulin from safflower seed meal was analyzed using an amino acid analyzer (S-433D, Sykam, Germany).

### 2.12 Statistical analysis

Each experiment was performed in at least triplicate, and all data are presented as mean ± standard deviation (SD). Statistical significance (*P* < 0.05) was determined using one-way ANOVA and Tukey test by SPSS Statistics 16.0 (IBM, USA).

## 3 Results and discussion

### 3.1 Proximate analysis of safflower seed meal

**Figure 1:**
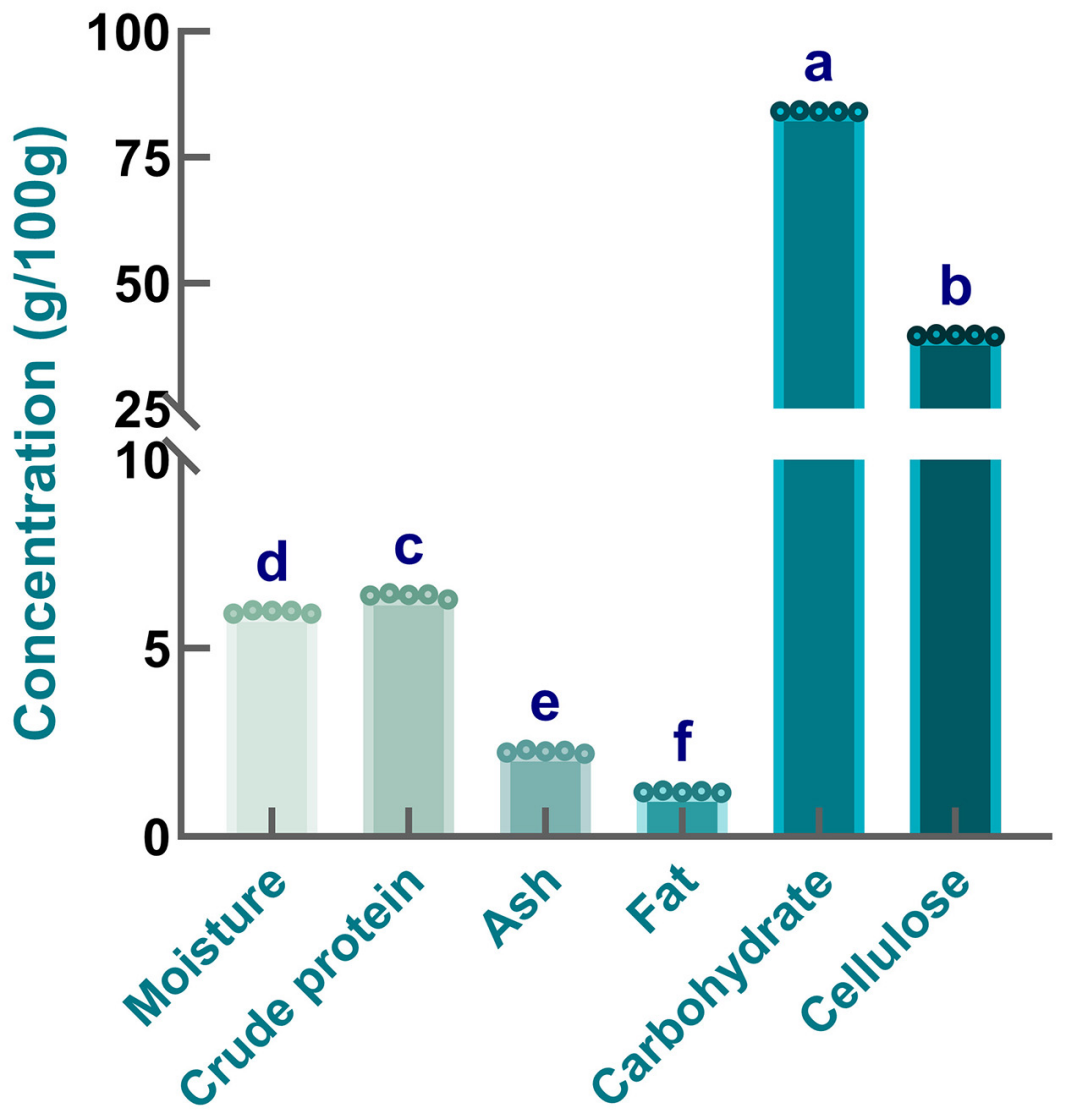
Proximate composition of safflower seed meal. Different letters indicate significant differences at *P* < 0.05.

To evaluate the nutritional profile of safflower seed meal, proximate analysis was conducted in the present work. As shown in Figure 1, the contents of carbohydrate, cellulose, crude protein, ash, fat, and moisture were 84.18 ± 0.11, 39.64 ± 0.19, 6.40 ± 0.06, 2.26 ± 0.04, 1.19 ± 0.02, and 5.96 ± 0.04 g/100 g, respectively. It was found that the moisture content of sample was relatively low, which was consistent with the moisture level reported for safflower seed (38). Additionally, compared with safflower seed meal, higher moisture levels were observed in several other plant seeds or seed meals, including defatted *Moringa oleifera* seed flour (39), annatto seed residue (40), perilla seed meal (41), and corn grain (42). Obviously, low moisture content is beneficial for minimizing the risk of microbial contamination and extending the storage life of safflower seed meal (43). When comparing the fat content of safflower seed meal with that of safflower seeds recorded by others (44), it is evident that a substantial amount of fat was removed during the oil extraction process. A low fat content is also particularly crucial for extending the shelf life of safflower seed meal. Generally, a high fat content may impair the functional properties of proteins, which is likely associated with protein-lipid interactions and an increased susceptibility of proteins to oxidative reactions (45). Additionally, ash content determination results indicated the presence of minerals at a certain concentration in samples. Specifically, the ash content of safflower seed meal was lower than that of seeds or cakes from other oil crops, including safflower (46), rapeseed, mustard (44), and soybean meal (47). These variations might be attributed to multiple factors, such as species-specific differences, growing season duration, soil type characteristics, and harvesting timing (48). Additionally, Figure 1 showed that cellulose, the predominant structural polysaccharide in cell walls, constituted the primary component of the carbohydrate fraction. This distribution pattern aligned with the characteristic non-starch polysaccharide profile observed in flaxseed (38). The high carbohydrate and cellulose content of safflower seed meal were likely attributable to the unprocessed state of the seeds (e.g., unhulled) (49). Moreover, it is worth noting that safflower seed meal still contained a large amount of proteins, even if this content was lower than that recorded by others (50). These differences might be explained by variations in variety or species, oil extraction technology, as well as differences in climatic conditions. Taken together, safflower seed meal provides candidate materials for the development of plant-derived bioactive substances.

### 3.2 The content and recovery rate of each protein fraction from safflower seed meal

**Figure 2:**
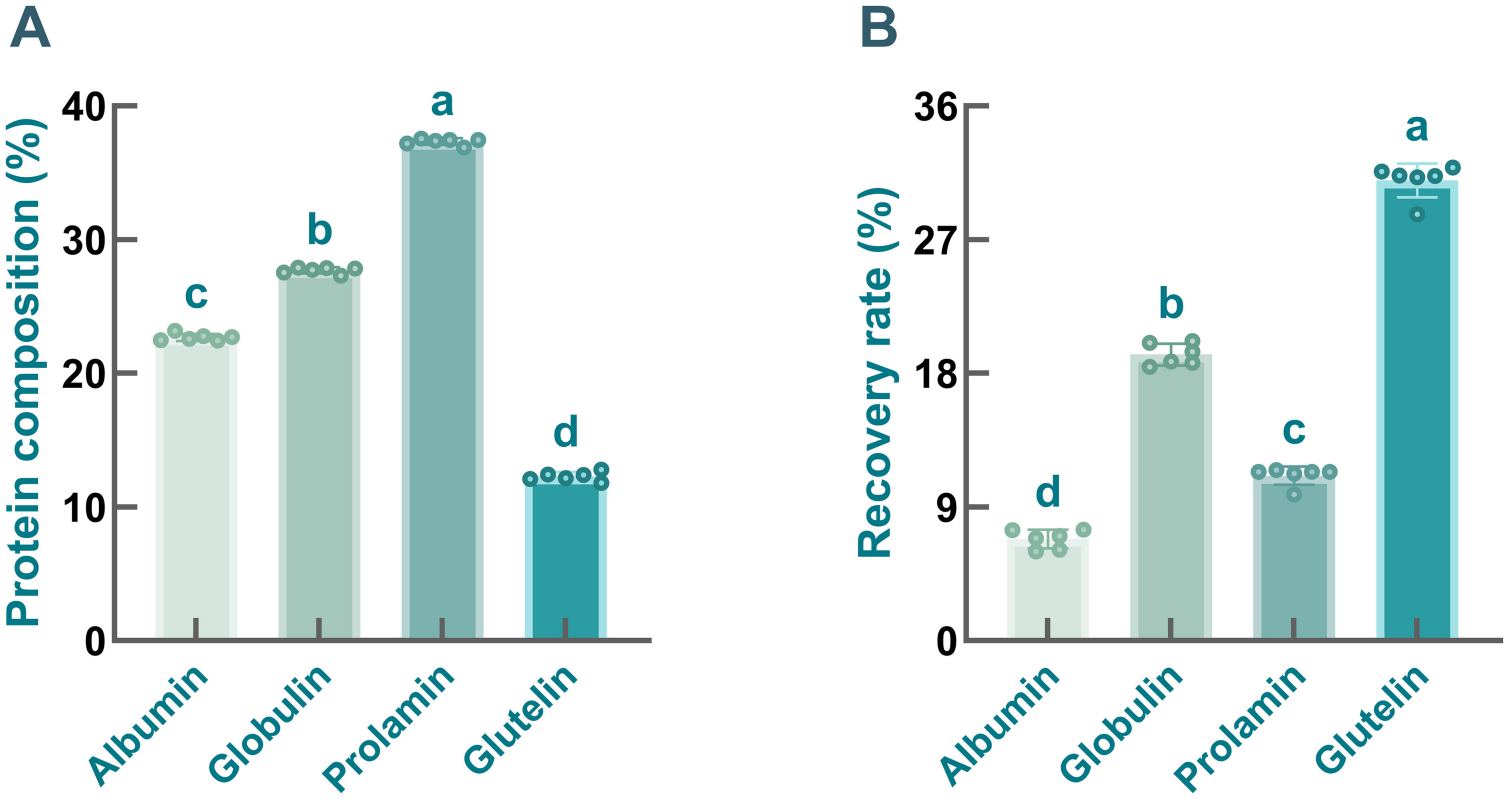
The contents **(A)** and recovery rates **(B)** of four protein fractions prepared from safflower meal. Different letters indicate significant differences at *P* < 0.05.

As mentioned above, the Osborne extraction method was employed to isolate albumin, globulin, prolamin, and glutelin from safflower seed meal, and their protein content and recovery rate were further determined. As illustrated in Figure 2A, the contents of albumin, globulin, prolamin, and glutelin in the samples were 22.69 ± 0.27%, 27.69 ± 0.23%, 37.33 ± 0.24%, and 12.28 ± 0.34%, respectively. Obviously, compared with glutelin, the contents of prolamin, globulin, and albumin in safflower seed meal were relatively higher, especially prolamin and globulin, which was consistent with previous studies. It has been documented that globulin, prolamin, and albumin account for a large proportion of storage proteins in many seeds (51). Nevertheless, differences in the content of protein fractions were found across different plant species (52). For example, in faba bean (Hud-line I) proteins, albumin and globulin were identified as the two most abundant fractions (53), whereas in pea proteins and sunflower proteins, globulin contents exceeded 55% (54). Shewry et al. (55) found that the storage proteins of corn (zein), sorghum (kafirin), and barley (hordein) were dominated by prolamin, followed by globulin. Additionally, some articles have noted the absence of prolamin in some plants, such as sugarbeets (51). However, in comparison of globulin, markedly lower recovery rates were found in prolamin-enriched and albumin-enriched fractions (Figure 2B, *P* < 0.001), which greatly limits their further application. Furthermore, numerous studies have demonstrated that plant-derived globulins possess additional advantages, including high availability, low production cost, and non-toxicity, making them an increasingly attractive research focus (54).

### 3.3 Functional properties of four protein fractions from safflower seed meal

As is well known, the functional characteristics of proteins are particularly important for their applications, among which solubility, surface hydrophobicity, emulsifying ability, oil- and water-holding capability, and foaming ability are the most important functional characters (56). Therefore, in the present work, the differences in various functional capacities of the four protein fractions derived from safflower seed meal were investigated.

#### 3.3.1 Solubility characteristics

**Figure 3:**
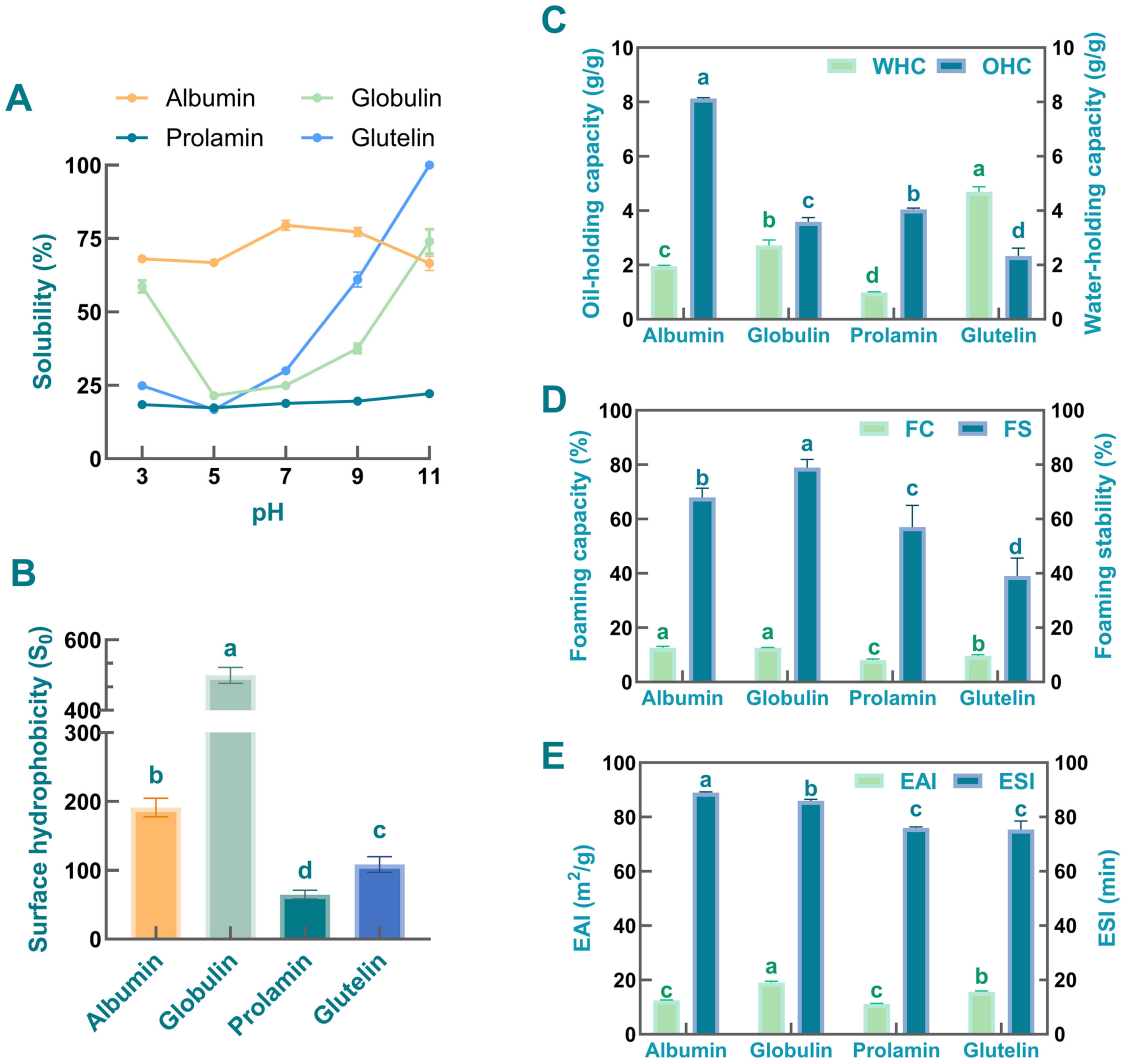
Functional characteristics of safflower seed meal protein fractions. **(A)** Solubility; **(B)** Surface hydrophobicity; **(C)** Oil- and water-holding capacity; **(D)** Foaming properties and foaming stability; **(E)** Emulsifying properties and foaming stability. Different letters indicate significant differences at *P* < 0.05. Values labeled with the same letter are not statistically different (*P* > 0.05).

Solubility is an important parameter for selecting the preparation methods of protein's subunits and understanding the potential applications of proteins (57). Moreover, it is well recognized that solubility plays a key role in regulating the emulsifying, foaming, and water-holding capacities of proteins (58). Therefore, the solubility characteristics of test proteins derived from safflower seed meal were first determined across a pH range of 3–11. As observed in Figure 3A, among these four protein fractions, albumin exhibited the highest solubility across all selected pH except pH 11. Consistent with this finding, compared with other fractions, higher solubility of albumins prepared from other samples [e.g., sesame bean (59), African yam bean seed (60), sunflower (61), and oat (62)] has also been reported at pH 3–9. It is speculated that the high aqueous solubility of albumin may be attributed to its amino acid composition and the limited exposure of hydrophobic residues on its protein surface (63). Meanwhile, as shown in Figure 3A, the prolamin-enriched fraction exhibited the lowest solubility at all pH values, likely due to its inherent hydrophobic nature in aqueous solutions (63). This result is consistent with previous findings by Adebiyi and Aluko. They found that the prolamin derived from pea seed exhibits low solubility in aqueous solutions (64).

Additionally, the solubility of the four protein fractions is predominantly regulated by the pH of the solutions (65). As the pH increased, the solubility of globulin and glutelin showed a U-shaped trend, with their minimum values observed at pH 5. This pattern mirrors the solubility behavior of globulin and glutelin derived from Semen Astragali Complanati (23) and commercial pea protein fractions (64). Moreover, the minimum solubility of prolamin and albumin was also observed at pH 5. It is speculated that the isoelectric point (PI) of these four fractions are close to pH 5, and these protein molecules usually aggregate through various non-covalent interactions at PI, thereby reducing their solubility (66). It was also noted that as the pH shifted toward alkaline conditions, the solubility of globulin and glutelin increased obviously, with both reaching their maximum values at pH 11.0. Glutelin was particularly notable, as it was almost completely dissolved at this pH. Referring to previous studies, the enhanced solubility under alkaline conditions can be attributed to two factors: (1) increased electrostatic repulsion due to higher surface charge density, and (2) enhanced hydration of ionized amino acid residues (67). However, albumin exhibited an unexpected solubility decline under strongly alkaline conditions (pH 11), likely due to its unique surface amino acid composition. The balanced surface distribution of hydrophobic and hydrophilic residues plays a critical role in governing protein solubility (68). Thus, it is hypothesized that beyond a specific pH threshold, excessive surface charge is likely to trigger protein conformational changes, exposing hydrophobic core regions and thereby reducing the protein's hydrophilicity (69). Based on these findings, a pH of 7 was selected for all subsequent experiments. This choice represented a balance between avoiding the reduced protein solubility (and consequent impairment of functional properties) at lower pH levels and the consumer unacceptability of highly alkaline food products.

#### 3.3.2 Surface hydrophobicity

Surface hydrophobicity is considered as a structural property crucial for the emulsification and foaming capacities of proteins (4). As presented in Figure 3B, it was observed that the globulin fraction exhibited the highest hydrophobicity (*S*_0_, 499.60), followed by albumin (191.20), glutelin (108.50), and prolamin (64.67). Previous studies indicate that variations in *S*_0_ among protein fractions are related to their hydrophobic residue content and the degree of exposure induced by processing or denaturation (70). Thus, the high surface hydrophobicity observed in the globulin fraction suggests a high density of exposed hydrophobic binding sites on its structures. This finding is consistent with reports on globulins from *Ginkgo biloba* seeds and rice, which also exhibit greater surface hydrophobicity than their corresponding glutelin fractions (25, 71). Notably, the greater surface hydrophobicity of globulin implies a potential for robust protein-protein hydrophobic interactions. As documented previously in wheat gluten protein studies, such interactions are likely to improve viscoelasticity (4). Results also showed that the *S*_0_ of albumin was significantly lower than that of globulin, which is might be associated with its higher solubility (4). A large number of studies have demonstrated that the *S*_0_ values of proteins are negatively correlated with their solubility (72). In general, an increase in hydrophobicity reduces the water solubility of protein molecules and may force these molecules to aggregate via hydrophobic interactions, ultimately decreasing their solubility in aqueous solutions (73). Compared with the above two protein fractions, the prolamin and glutelin fractions exhibited relatively lower surface hydrophobicity. As supported by previous studies (70, 74), this aggregation entraps hydrophobic regions within the protein's interior during the initial aqueous solubilization, thereby shielding them from detection.

#### 3.3.3 Oil-holding and water-holding capacity

Protein hydration capacity, commonly referred to as WHC, represents protein's ability to bind and retain water within its structural matrix. This functional property plays a pivotal role in maintaining moisture stability during food processing and storage (75). Conversely, OHC characterizes the lipid-binding properties of proteins, which significantly influences the sensory attributes, textural properties, and fat retention efficiency of final products (76). Figure 3C demonstrates significant variations in WHC among four distinct protein fractions isolated from safflower seed meal, which might be associated with differences in their intrinsic properties, including molecular weight and intermolecular interaction (77). Specifically, glutelin exhibited the highest water retention capacity (4.70 ± 0.18 g/g), followed sequentially by globulin (2.83 ± 0.01 g/g) and albumin (1.96 ± 0.03 g/g), while prolamin showed relatively low performance (0.99 ± 0.03 g/g). The water-holding capacity of glutelin obtained in this work was slightly lower than that of soy protein isolate (26). Nevertheless, its WHC remained higher than that of protein isolates from other plant sources, including blackberry, sweet pepper seed, red bean, and peanut (26). Notably, as documented in other studies, prolamin fraction prepared from *Rana chensinensis* ovum also exhibited lowest water-holding capacity (78). In terms of globulins, the globulins from some samples were also found to have extremely low water-holding capacity, which was different from the results obtained in this work (26). On the contrary, the globulins extracted from *Ginkgo biloba* seeds and Semen Astragali Complanati exhibited high WHC, sharing similar characteristics with the safflower seed meal globulin (23, 25). The high abundance of hydrophilic amino acid residues in globulin molecules is likely a pivotal structural factor contributing to their superior water-holding capacity (69). It is widely accepted that an appropriate WHC is particularly important for viscous foods (gravies, baked products, etc.). For instance, prior studies have proposed the WHC of peanut protein applied in sticky food as 1.49–4.72 g/g. Hence, four obtained protein fractions are applicable for utilization in this category of food products (26).

Regarding oil-holding capacity, the four protein fractions exhibited distinct oil retention capabilities, ranking in descending order as: albumin (8.13 ± 0.03 g/g) > prolamin (4.05 ± 0.05 g/g) > globulin (3.59 ± 0.16 g/g) > glutelin (2.33 ± 0.29 g/g). These differences in oil-holding capacity among the protein fractions of safflower seed meal may be related to their conformational properties, surface charge, surface hydrophobicity, and lipophilic groups (25, 26). Among the four protein fractions isolated from safflower seed meal, albumin exhibited significantly higher OHC than the other three components, with performance comparable to that from African yam bean seeds (60). Similarly, previous studies have reported strong oil absorbing capacity in albumins prepared from American locust bean (79), kidney beans (80), blackberry (26), banana peel powder (81). Based on previous studies, abundant surface-exposed hydrophobic residues may be one of the reasons contributing to the high oil-holding capacity of the albumin fraction, as these residues facilitate stronger protein-lipid interactions via hydrophobic effects (60). The high OHC of albumin indicates that this protein holds promise as an ingredient in food products such as salad dressings and sausages.

#### 3.3.4 Foaming property

Foaming property is widely recognized as one of the important functional properties of proteins. Accumulated evidence has revealed that high-speed shearing generates a strong physical impact, which disrupts the secondary structure of protein, exposes its hydrophobic groups, and ultimately enables air to stably incorporate into the liquid to form bubbles (82). The foaming property of proteins directly affects their applicability in food systems (83). Therefore, the FC and FS of the four protein isolates were also determined. As presented in Figure 3D, the globulin- and albumin- enriched fractions exhibited significantly higher FC than the prolamin and glutelin samples (*P* < 0.05). Meanwhile, no obvious difference in FC was observed between globulin and albumin (*P* > 0.05). The difference in FC values among the four protein fractions may be attributed to the variation in their surface hydrophobicity. A growing body of evidence indicates that higher surface hydrophobicity of protein molecules leads to greater interfacial adsorption at the air-water interface, lower interfacial tension, and improved bubble stability. These effects collectively represent one of the potential mechanisms underlying the excellent foaming performance of globulin and albumin (84). A positive correlation between protein solubility and foaming capacity has been reported in previous studies (85). In general, this finding is consistent with the results of solubility and foaming capacity measurements in the present study.

With respect to FS, obviously differences were found among the four protein fractions, with globulin exhibiting a significantly higher FS than the other three protein fractions (Figure 3D, *P* < 0.05). This finding demonstrates the globulin's capacity to sustain foam stability for an extended period. Previous studies have reported that the foaming stability of proteins is not only associated with their solubility, but also influenced by many factors, such as protein hydrophobicity, visco-elasticity, etc. (37). Thus, these factors might affect the foaming stability of four protein fractions. Taken together, comparative analysis of the differences in FC and FS leads to the conclusion that safflower seed meal derived-globulin exhibits excellent foaming feature.

#### 3.3.5 Emulsifying property

Accumulated evidence has revealed that plant-derived proteins are increasingly recognized as effective natural emulsifiers in various applications (86). In the present study, to investigate the emulsifying features of four protein fractions, EAI and ESI were calculated, which are commonly used indicators for assessing the emulsifying activity and emulsifying stability of proteins (87). As represented in Figure 3E, among the tested samples, the globulin exhibited prominent emulsifying ability, due to its highest EAI, which was higher than that of hemp seed protein (88). The strong emulsifying capacity of globulin may be related to its high surface hydrophobicity. Specifically, a rise in surface hydrophobicity facilitates the adsorption of proteins onto the oil-water interface, lowers surface tension, and ultimately improves the proteins' emulsifying performance (74). Additionally, results showed that the ESI of globulin (85.96 ± 0.61 min) was only lower than that of albumin (88.94 ± 0.40 min) but significantly higher than those of prolamin (75.98 ± 0.38 min) and glutelin (75.37 ± 3.17 min). These data indicate that the emulsifying capacities of globulin and albumin derived from safflower seed meal were superior to those of the other two protein fractions. This phenomenon is presumably due to the ability of globulin and albumin to generate stronger electrostatic repulsion, which reduces aggregation between oil droplets and thereby preventing emulsion from breakage (23). Additionally, markedly higher ESI was found in safflower seed meal than those in pea protein isolate (89), lotus seed protein isolate (90), rice bran protein (91), soybean protein isolates (92), wheat germ protein (93), etc.

These findings show that safflower seed meal proteins have functional properties, enabling their practical application as ingredients in the food industry. After a comprehensive analysis of these parameters, particular attention was paid to the globulin and albumin. However, the relatively low content and recovery rate of albumin also constitute limiting factors that hinder its application. Taken together, considering the functional performance, extraction efficiency, and economic feasibility, globulin was selected for subsequent experiments.

### 3.4 Impacts of different parameters on the extraction efficiency of globulin from safflower seed meal

To investigate the effects of four tested parameters on the extraction yield of globulin, single-factor tests were employed here.

#### 3.4.1 Extraction time

**Figure 4:**
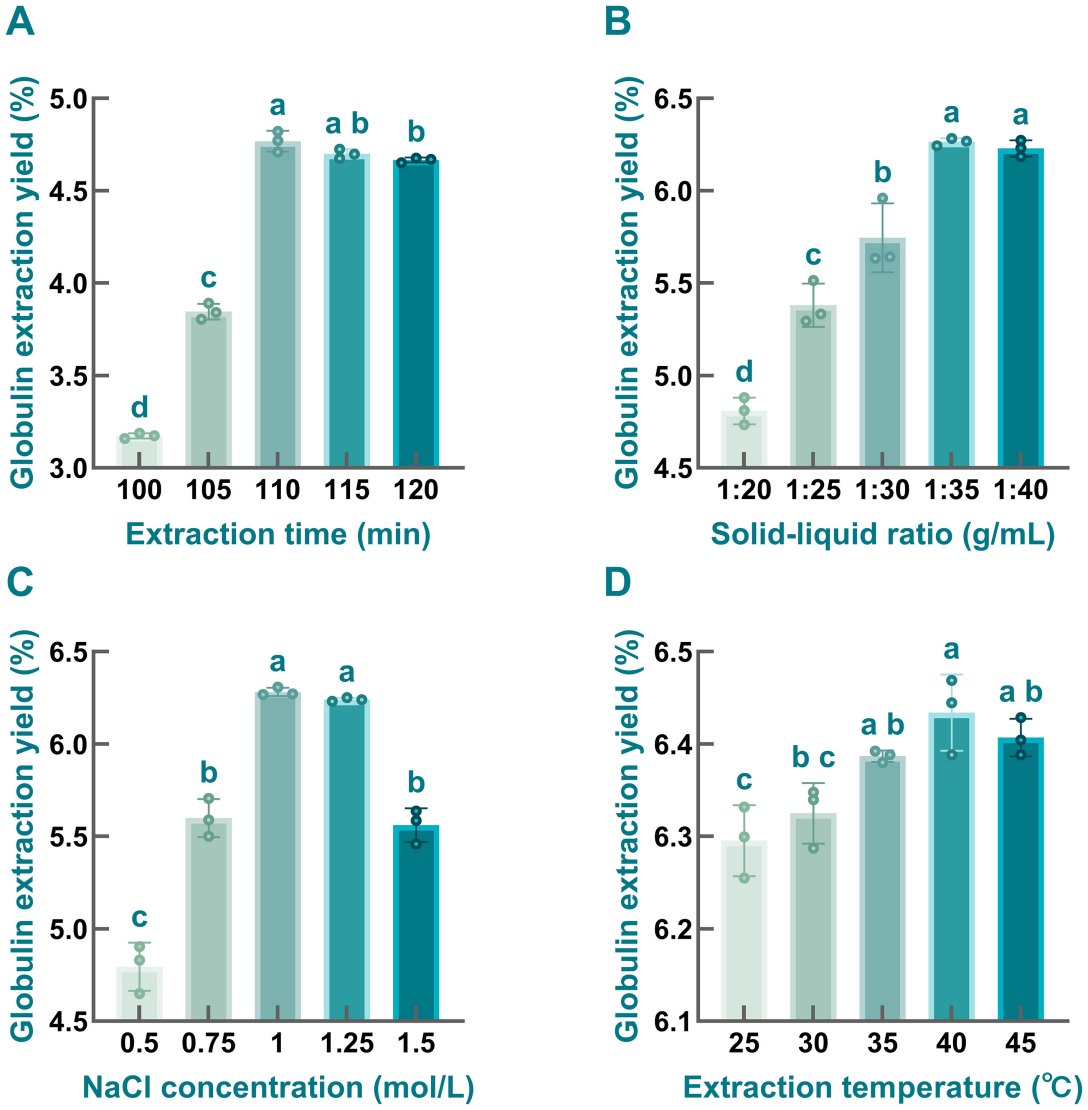
The impacts of extraction parameters on the globulin yield prepared from safflower seed meal. **(A)** extraction time; **(B)** solid-liquid ratio; **(C)** NaCl concentration; **(D)** extraction temperature. Different letters indicate significant differences at *P* < 0.05. Values labeled with the same letter are not statistically different (*P* > 0.05).

As depicted in Figure 4A, the extraction yield of globulin first increased and then slightly decreased with the extension of extraction time, reaching its peak at 110 min. Notably, significant differences (*P* < 0.05) were observed between the 110-min treatment and other groups, except for the 115-min group. This trend is consistent with findings from previous studies. For instance, Zhao and coworkers found that the extraction efficiency of 7S globulin from soybean initially increased and subsequently decreased with prolonged extraction time, regardless of the α, α', or β subunits (94). Prolonged extraction time enhances globulin yield by strengthening protein-solvent interactions, which in turn facilitates molecular diffusion (95). However, protein molecules may undergo partial denaturation under prolonged salt ions conditions, resulting in protein aggregation into insoluble proteins that were removed, leading to prolonged extraction ultimately reducing the efficiency of globulin extraction (96). Therefore, 110 min was identified as the optimal extraction time for subsequent experiments.

#### 3.4.2 Solid-liquid ratio

As illustrated in Figure 4B, when the solid-liquid ratio increased from 1:20 to 1:35 g/mL, the extraction yield of globulin was enhanced, with marked differences observed among the tested solid-liquid ratios. However, further increasing the proportion of extraction solvents did not significantly improve the extraction yield of the target protein; instead, a slight reduction was noted. This trend is consistent with observations reported in previous studies (26). Therefore, a solid-liquid ratio of 1:35 g/mL was selected as optimal, as it yielded the highest globulin extraction yield.

#### 3.4.3 NaCl concentration

Considering the salting-in and salting-out effects, the concentration of salt solution is usually considered as a key factor influencing protein extraction efficiency (97). In the present study, the globulin yield initially increased and then decreased with rising NaCl concentration, with the maximum yield (6.28 ± 0.02%) observed at a NaCl concentration of 1 mol/L (Figure 4C). This finding was in line with the previous investigation carried out by Mao and Hua (98). As a salt-soluble protein, globulin extraction can be enhanced by increasing NaCl concentration to a certain extent, which may be attributed to the effect of salt-in effect (97, 99). However, once the NaCl concentration exceeded 1 mol/L, the extraction yield of globulin gradually declined, which likely resulted from salt-induced precipitation (96). In addition, the competitive binding between excessive salt ions and charged molecules of globulin to water from the extraction solvent may also be another reason for this decline. In the other words, elevated salt concentration may reduce the interaction between protein and water molecules, further weaken protein hydration, and strengthen the hydrophobic interactions, ultimately leading to protein aggregation and precipitation (96, 100). Therefore, 1 mol/L NaCl was selected as the optimal concentration for subsequent experiments.

#### 3.4.4 Extraction temperature

As noted in Figure 4D, the highest extraction yield of globulin was found in the 40 °C treatment group, whereas yields decreased to varying extents at temperatures below or above 40 °C. Generally, a positive correlation existed between temperature and globulin extraction efficiency within the temperature range of 25–40 °C, indicating that increasing the temperature within a certain range facilitates protein release from the samples. This phenomenon may be attributed to the fact that elevated temperature may alter the three-dimensional structure of globulin, making it more extended and thereby enhancing its contact with the extraction solvent, ultimately improving globulin extraction efficiency. However, excessive high extraction temperatures may cause globulin aggregation, which is unfavorable for increasing the yield of target protein (26). Consequently, the optimal extraction temperature was set at 40 °C.

### 3.5 Determining the proper values of four tested factors

BBD was introduced to further find the suitable values of four factors, and the results are displayed in Table 1. Clearly, the extraction yield of globulin varied markedly under different extraction conditions, with values spanning from 2.96% (minimum) to 6.80% (maximum). This suggests that establishing the optimal conditions for globulin extraction is particularly necessary. Two models were employed to fit the experimental data.

#### 3.5.1 Optimization using RSM

As mentioned above, the MQR model shown below was established to explain the relationship between factors and response value.


$$\begin{array}{c} Y\;=\;41.9483\;+\;0.5361A\;+\;0.0773B\;+\;0.6195C\;+\;5.8267D\\ \;+0.0028AB\;0.0049AC\;0.0004AD\;0.0011BC\;+\;0.0067BD \end{array}$$

$$\begin{array}{c} \;+0.0134CD-\;0.0020A^{2}\;0.0039B^{2}\;0.0004C2\;2.8710D^{2} \end{array}$$ (7)

Furthermore, the ANOVA results were depicted in Table 2. Results revealed that a significant model was obtained in the present work, supported by its high *F*-value (14.20, the bigger the better) and low *P*-value (*P* < 0.001, the smaller the better). Additionally, non-significant lack of fit (*P* > 0.05) was also determined, further indicating the satisfactory predictive ability of this model. The *R*^2^ (0.93) and adjusted *R*^2^ (0.87) were both higher than 0.8, which was the threshold of an ideal model, suggesting that the MQR model is suitable for describing the dependence of globulin yield on these four variables (95).

**Table 2:** Analysis of variance for MQR model.

**Source**	**Sum of squares**	**df**	**Mean square**	***F*-value**	***P*-value**
Model	29.21	14	2.09	14.20	< 0.0001
*A*-time	0.03	1	0.03	0.20	0.66
*B*-solid-liquid ratio	17.31	1	17.31	117.80	< 0.0001
*C*-temperature	0.76	1	0.76	5.15	0.04
*D*-NaCl concentration	1.98	1	1.98	13.45	0.0025
*AB*	0.73	1	0.73	4.94	0.04
*AC*	0.95	1	0.95	6.44	0.02
*AD*	0	1	0	0.0001	0.99
*BC*	0.11	1	0.11	0.76	0.40
*BD*	0.01	1	0.01	0.07	0.80
*CD*	0.02	1	0.02	0.12	0.73
*A* ^2^	0.27	1	0.27	1.81	0.20
*B* ^2^	4.96	1	4.96	33.74	< 0.0001
*C* ^2^	0.01	1	0.01	0.08	0.78
*D* ^2^	3.34	1	3.34	22.74	0.0003
Residual	2.06	14	0.15		
Lack of fit	1.64	10	0.16	1.56	0.36
Pure error	0.42	4	0.11		
Cor total	31.27	28			

**Figure 5:**
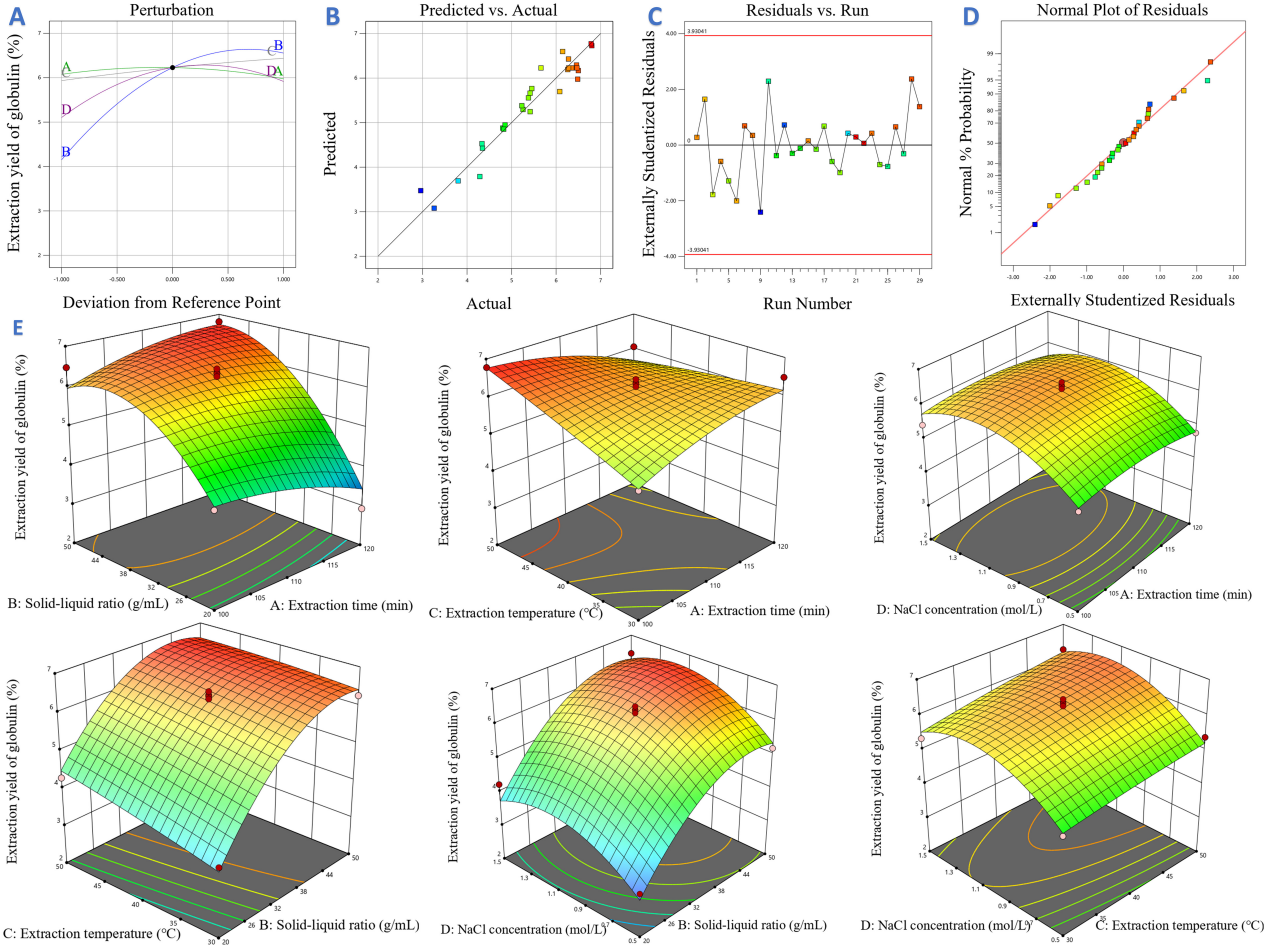
The results of the response surface analysis. **(A)** Perturbation plot; **(B)** Plots of predicted and actual values; **(C)** Diagram of residuals vs. number of runs; **(D)** Normal probability distribution; **(E)** Response surface plots and contour plots.

Additionally, multiple diagnostic tools were employed to assess the reliability of the MQR model. As shown in the perturbation plot (Figure 5A), the relative impacts of different parameters on the response variable exhibited marked variability. Specifically, the nearly horizontal response curve of factor *A* indicated its minimal impact on extraction efficiency of target protein. In contrast, factors *C, B* and *D* exhibited distinct slope variations, reflecting their substantial regulatory effects on the response. These observations were consistent with the ANOVA results presented in Table 2, where variables *B, C, D* showed statistically significant effects, confirming their deterministic roles in the globulin extraction process. In Figure 5B, the high agreement was observed between the predicted value and experimental data, as evidenced by the close clustering of data points. This data indicates the good fitness of the equation. Furthermore, to check the potential variables that affect the response variable during the experiment, the run order plot of studentized residuals was obtained (Figure 5C). Results showed that no outliers beyond the confidence limits (red lines), indicating that all data points exhibited acceptable error margins. Additionally, the distribution of residuals in Figure 5C did not show any special patterns. Figure 5D displays the normal probability plot of residuals, which is commonly used to know whether the difference between the experimental data and predicted data follow a normal distribution (101). In the present work, a linear distribution of residuals was found, demonstrating the validity of the model. Collectively, these observations further validate the reliability and adequacy of the MQR model for predicting the optimal globulin extraction conditions.

Based on the data presented in Table 2, the *P*-value of linear terms (*B, C, D)*, quadratic terms (*B*^2^ and *D*^2^) were all lower than 0.05, indicating that the extraction yield of globulin was markedly influenced by these terms. Among them, all linear terms contributed positively to the response value, while the quadratic terms had negative contributions. The relative impacts of variables on the extraction efficiency of globulin from safflower seed meal ranked as *B* > *D* > *C* > *A*. For interactive effects, the *AB* term showed positive influence on globulin extraction efficiency, whereas *AC* exhibited a negative effect. Furthermore, the response surface and contour plots were drawn with the help of Design-Expert13 software, which were consistent with the ANOVA results (Figure 5E).

The maximum extraction yield of globulin was predicted to be 7.03% (95% confidence interval: 6.08%−7.98%) using the optimal extraction parameters (extraction time of 110 min, solid-liquid ratio of 1:50 g/mL, extraction temperature of 33 °C, and NaCl concentration of 1.24 mol/L) predicted by RSM. To validate the reliability of this prediction, five replicate globulin extraction experiments were conducted, yielding an average globulin extraction yield of 6.93 ± 0.12%. Experimental results showed a relative deviation of 1.42% from model predicted value. The strong consistency between predicted and experimental values conclusively confirms the predictive accuracy and reliability of the MQR model.

#### 3.5.2 ANN-GA optimization

**Figure 6:**
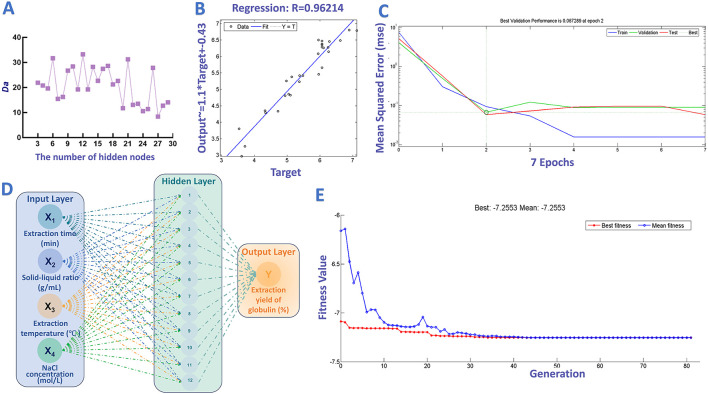
ANN-GA-based optimization of globulin extraction process. **(A)** Effect of different hidden neurons on the degree of approximation; **(B)** Results of regression analysis; **(C)** Training performance of ANN model; **(D)** Architecture topology of ANN model established in this work. **(E)** The change in fitness value with the increase in generation.

In addition to RSM, ANN-GA was also introduced to fit and optimize the extraction parameters for globulin preparation. This intelligent technique has gained growing attention due to its superior predictive accuracy and optimization efficiency (102). In this study, a three-layer ANN (input, hidden, output) was used to model the variable-yield relationship for globulin extraction. As mentioned above, selecting suitable hidden neurons of the ANN model is crucial for the fitting performance of the ANN model. As shown in Figures 6A, B, the proper number of hidden neurons was 12, due to its maximum *Da* (33.25), and high correlation coefficients (*R* > 0.96). Additionally, Figure 6C shows variation of mean squared error (MSE) vs. iteration. Following 2nd iterations, ANN model with 12 hidden neurons showed lower MSE (0.0673). Consequently, the topological architecture of ANN model with best-performance was established and identified as 4-12-1 (Figure 6D).

Afterward, GA tool was introduced to optimize the proper values of selected factors referring other studies. As illustrated in Figure 6E, increasing the number of generations led to a gradual decrease in the fitness value, which reached a near-steady state after the 44th generation. After optimization, the proper extraction time, solid-liquid ratio, NaCl concentration and extraction temperature were determined to be 110 min, 1:47 g/mL, 1.24 mol/L and 37 °C, respectively. Under the above conditions, the maximum extraction yield of globulin was expected to reach 7.26%. Similarly, to verify this prediction, globulin extraction was carried out under the predicted conditions, and the average extraction ratio of target protein obtained from five validation experiments was 7.33 ± 0.10%. The deviation between the predicted maximum response value and experimental results was small (0.96%), indicating the acceptability of the model's predictions. Furthermore, the ANN-GA approach increased the globulin yield by 130.96% compared to the yield obtained before optimization. Of course, further improving the extraction yield of safflower seed meal globulin at the industrial scale remains a key focus of our future research.

The predictive performances of the RSM and ANN models were assessed and compared using parameters of RMSE, MAE, *R*^2^, AARD, and ARE (Supplementary Table S1). The high *R*^2^ values of both models (RSM: 0.93, ANN: 0.90) demonstrate their adequacy in representing the experimental results. However, compared to ANN model, RSM model exhibited lower RMSE (RSM: 0.27, ANN: 0.29) and MSE (RSM: 0.07, ANN: 0.08), AARD (RSM: 4.17, ANN: 4.71), and ARE (RSM: 4.17, ANN: 5.69). These findings suggest that compared to the ANN model, the RSM model exhibits high robustness and reliability in simulating the relationship between the four test factors and globulin production. The predictive performance of a model is influenced by factors such as the number of experiments and process complexity. While ANN is applicable to both small and large datasets, RSM often demonstrates superior performance in constrained settings (103). In this study, with only 29 experimental data points, the RSM model yielded better results. Furthermore, the ANN framework cannot be used to elucidate the individual and interactive effects among the independent variables (104). This finding is consistent with results from numerous previous studies (70, 105–109). In summary, RSM demonstrates superior predictive accuracy, whereas ANN provides a higher theoretical maximum within the design space.

### 3.6 Analyzing the molecular weight and amino acid composition of globulin from safflower seed meal

The molecular weight and amino acid composition of globulin from safflower seed meal were further detected. As depicted in Table 3, a total of 17 amino acids were identified in the globulin sample, including 8 essential amino acids (EAAs) and 9 non-essential amino acids. Quantitative analysis revealed that the EAAs accounted for 26.09% of the total amino acids, a proportion higher than rapeseed meal protein (110). Notably, the globulin not only contained most EAAs required by humans, but also showed significantly higher levels of each EAA compared to the adult amino acid intake standards recommended by FAO/WHO (111). Among the quantified amino acids, glutamate (17.12 ± 0.15 g/100 g) and proline (18.69 ± 0.20 g/100 g) were the predominant components in the globulin fraction. This amino acid profile was highly similar to that of wheat gluten (112). It is recorded that the proteins with elevated glutamate content can enhance immune response and modulate central nervous system activity. Furthermore, glutamate exhibits potent antioxidant properties, due to its ability to donate excess electrons, thereby neutralizing free radicals (113). Regarding proline, its high proline content, along with other hydrophobic amino acids, promotes the compact folding of the protein core via hydrophobic interactions and van der Waals forces, thereby effectively suppressing protein aggregation (114). Additionally, a rich proline content combined with other hydrophobic amino acids has been reported to be beneficial for reducing certain health risks (115). Additionally, the hydrophobic amino acids content was 46.88%. Overall, this evidence further provides possibilities for application of globulin derived from safflower seed meal in the food and pharmaceutical industries.

**Table 3:** Amino acid composition of globulin prepared from safflower seed meal.

**Amino acids**		**Contents (g/100 g)**	**FAO/WHO for adult**
Essential amino acids	Histidine	3.25 ± 0.07	1.60
	Methionine	0.83 ± 0.01	
	Lysine	1.72 ± 0.02	1.60
	Leucine	5.71 ± 0.06	1.90
	Isoleucine	3.27 ± 0.04	1.30
	Threonine	2.61 ± 0.02	0.90
	Valine	4.31 ± 0.05	1.30
	Phenylalanin	4.35 ± 0.05	
	Total	26.09 ± 0.23	
Non-essential amino acids	Aspartic acid	9.86 ± 0.09	
	Serine	3.14 ± 0.04	
	Glutamate	17.12 ± 0.15	
	Glycine	3.90 ± 0.05	
	Alanine	3.98 ± 0.03	
	Cystine	2.52 ± 0.05	
	Tyrosine	3.10 ± 0.05	
	Arginine	9.17 ± 0.10	
	Proline	18.69 ± 0.20	
	Total	71.47 ± 0.77	

**Figure 7:**
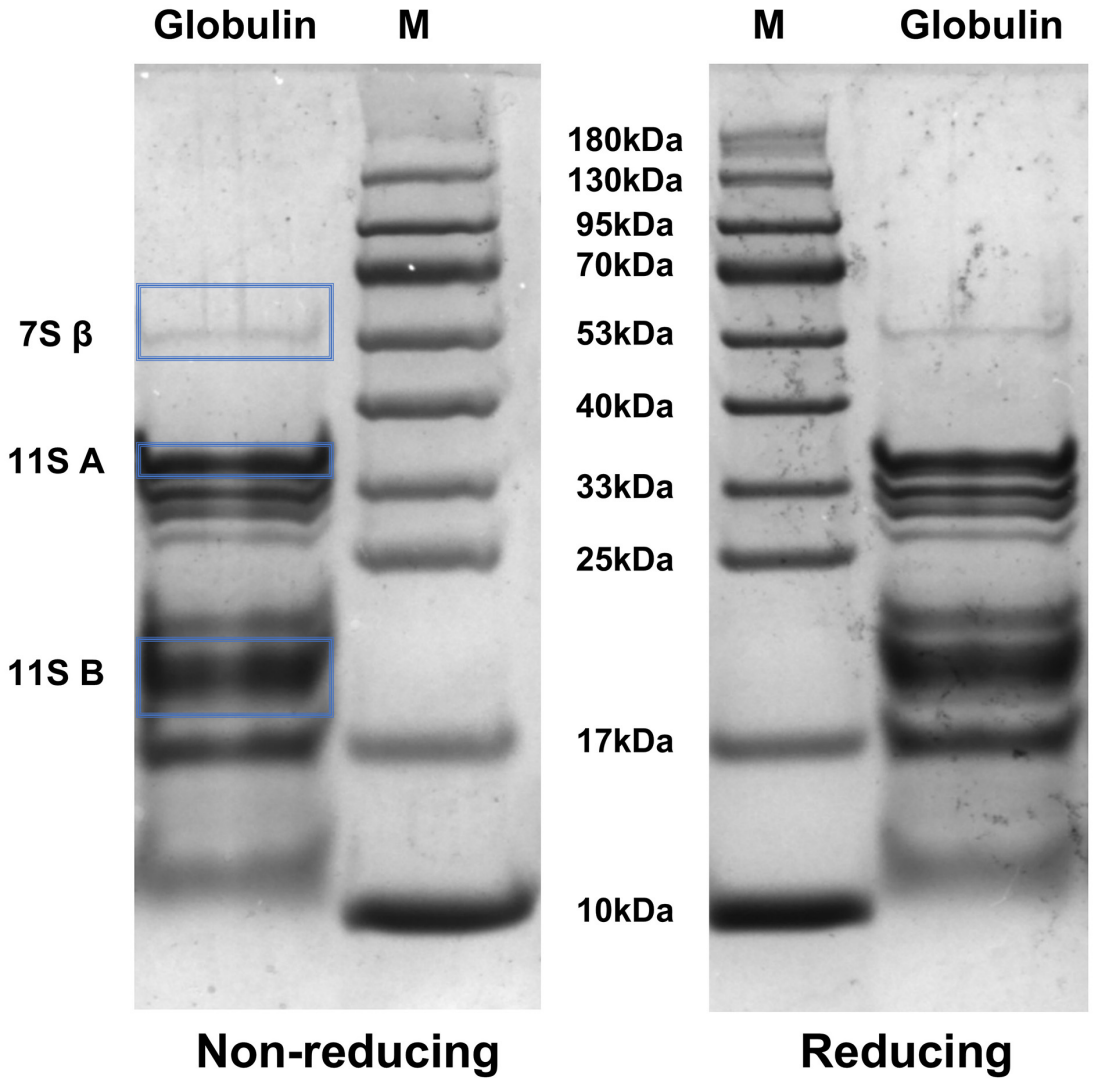
Molecular weight distribution of globulin extracted from safflower seed meal under reducing and non-reducing conditions. M: protein marker (10–180 kDa), Globulin: globulin from safflower seed meal. β: β-subunit of the 7S globulin; A: acidic subunit of the 11S globulin; B: basic subunit of the 11S globulin.

Figure 7 presents the electrophoretic profile of safflower seed meal derived globulin under both reducing and non-reducing conditions. Under non-reducing condition, the molecular weight of globulin was roughly distributed between 13 kDa and 53 kDa, among which several bands with molecular weights between 17 kDa and 40 kDa are particularly prominent, indicating that these protein subunits with these molecular weights have a high content in the sample. These findings show high concordance with the mass spectrometry profile of safflower seed proteins reported by Nosheen et al. (116). Meanwhile, the protein bands near 13 kDa and 53 kDa exhibited relative low intensity. These findings reveal the presence of abundant low-molecular-weight polypeptides (< 100 kDa) in the globulin sample. Previous studies have reported that low-molecular-weight polypeptides generally exhibit superior interfacial properties relative to high-molecular-weight proteins. Their presence is also associated with improved emulsion quality and the formation of smaller oil droplets (117). Furthermore, the smaller oil droplets stabilized by these low-molecular-weight polypeptides can enhance resistance to gravity-induced phase separation, thereby improves the overall emulsion stability (4). This might be aligned well with the excellent emulsifying stability observed in safflower seed meal globulin in the present study.

Based on differences in sedimentation coefficients, globulins can be primarily classified into two major categories: 7S globulins (including vicilin/convicilin) and 11S globulins (118). Specifically, 7S globulins typically exist as trimers with an apparent molecular weight of approximately 150 kDa; these trimers are composed of 50 kDa monomers, which can further dissociate into smaller subunits (α', α, and β) (119). In contrast, 11S globulins form hexameric structures (360–400 kDa), with each 60 kDa monomer consisting of a 40 kDa acidic Lα subunit and a 20 kDa basic Lβ subunit connected by disulfide bonds (120). SDS-PAGE analysis of globulin extracted from safflower seed meal revealed the presence of a 7S globulin β-subunit with an apparent molecular weight of 53 kDa. Additionally, bands corresponding to approximately 35 kDa and 20 kDa were identified as the acidic and basic subunits of 11S globulin, respectively.

Notably, despite the ability of the reducing agent β-mercaptoethanol to selectively cleave disulfide bonds, globulin exhibited remarkable structural stability under reducing conditions, with only minor alterations (121). A similar observation was reported for the protein of Chinese quince (*Pseudocydonia sinensis*) by Deng et al. (122). This result suggests that safflower seed meal globulin contains relatively low levels of disulfide bonds. Collectively, these findings not only provide deeper insights into the protein composition of safflower seed meal but also lay a crucial foundation for subsequent proteomic investigations.

### 3.7 FTIR detection of globulin from safflower seed meal

**Figure 8:**
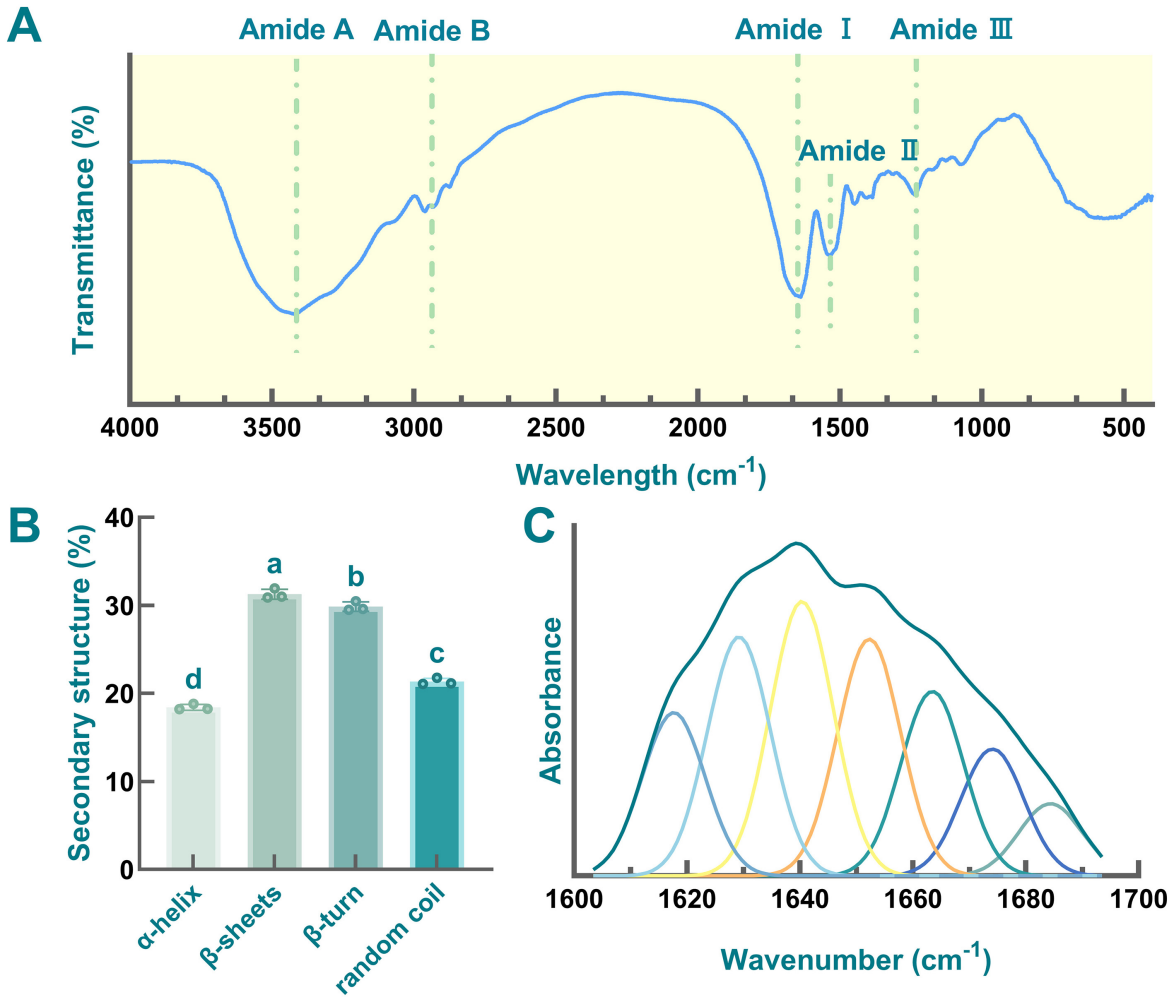
FTIR detection of globulin from safflower seed meal. **(A)** Fourier-transform infrared spectroscopy; **(B)** Secondary structure; **(C)** Curve-fitting spectra of globulin from safflower seed meal in the amide I region. Different letters indicate significant differences at *P* < 0.05.

FTIR spectroscopic analysis was employed to elucidate the characteristic functional group vibrations and secondary structural features of globulin derived from safflower seed meal (Figure 8A). As mentioned in previous studies, the FTIR spectra of proteins are typically dominated by amide bands, including amide A (3,500–3,300 cm^−1^), amide B (3,200–3,100 cm^−1^), amide I (1,700–1,600 cm^−1^), amide II (1,480–1,575 cm^−1^), and amide III (1,220–1,330 cm^−1^). These bands arise from the vibrations of the peptide segments within the proteins (74, 123). In this work, a broad absorption band was observed in the range of 3,500–3,300 cm^−1^ (amide A region), which was primarily attributed to the stretching vibrations of N-H and O-H bonds. This band is a characteristic feature associated with hydrogen bonding in the peptide backbone (124). Additionally, the signals in the region of 3,000–2,900 cm^−1^ were assigned to amide B. Moreover, distinct peaks between 1,657 and 1,240 cm^−1^ confirmed the presence of amide I, II, and III bands (23). As shown in Figure 8A, the observed amide III bands (≈ 1,220–1,350 cm^−1^) and amide II (≈ 1,480–1,575 cm^−1^) were primarily due to the C-N stretching vibrations and N-H bending vibrations in globulin, respectively (125). Additionally, amide I region was considered to provide the most information for understanding the secondary structure of target protein, consisting of several bands (126). Here, the absorption peaks observed between 1,600 and 1,700 cm^−1^ were assigned to the amide I region (1,600–1,700 cm^−1^), which was primarily attributed to C=O stretching vibrations (127). Furthermore, several adsorption bands in the amide I region were identified, corresponding to characteristic secondary structure elements, including α-helix (1,660–1,650 cm^−1^), β-sheet (1,640–1,610 cm^−1^), β-turn (1,700–1,660 cm^−1^), and random coil (1,650–1,640 cm^−1^), respectively (128).

The quantitative analysis of the secondary structures and curve-fitting spectra of the amide I region were shown in Figures 8B, C, respectively. The secondary structure of globulin was predominantly composed of β-sheets (30%) and β-turn (29%), with a composition ratio similar to that of other legume vicilin-type proteins, such as phaseolin from French bean, vicilin from pea, and β-conglycinin from soybean (129–131). However, compared to soybean 7S globulin, safflower seed meal globulin exhibited a significantly higher proportion of random coil (21%) (119), indicating the high flexibility of the globulin (132). Additionally, proteins with elevated random coil content have been reported to show increased susceptibility to digestion (66). Furthermore, the α-helix content (18%) was markedly lower than that of other fractions (*P* < 0.05), while the β-sheets content was the highest, consistent with previous study (74). Most plant globulins exhibit lower α-helix content, along with abundant β-sheets and random coil (133). Moreover, a negative correlation between α-helix content and hydrophobicity has been reported (124). Therefore, the low α-helix content in safflower globulin may also contribute to its enhanced hydrophobicity.

## 4 Conclusions

This study successfully isolated four protein fractions (albumin, globulin, prolamin, and glutelin) from safflower seed meal via the Osborne extraction method. Among these fractions, the globulin fraction exhibited the most promising development potential due to its superior performance, including relatively high content, favorable recovery rate, excellent foaming properties, and strong emulsion stability. Furthermore, intelligent optimization methods were employed to determine the optimal extraction parameters for globulin. Under these optimized conditions, a maximum globulin yield of 7.33 ± 0.10% was achieved. Structural characterization revealed that the globulin was characterized by small molecular weights (13–53 kDa), and β-sheet and β-turns were its predominant conformations. Additionally, amino acid analysis identified 17 amino acids in the globulin, including 8 essential amino acids, with hydrophobic amino acids accounting for 46.88%. Collectively, these results provide deeper insights into the properties of safflower seed meal globulin, thereby facilitating its innovative application in nutrition- and health-focused food formulations. However, further research is required to verify the feasibility of safflower seed meal globulin for industrial-scale production. Additionally, its health benefits, digestibility, oral safety, and specific applications in food products also merit further investigation.

## Data Availability

The original contributions presented in the study are included in the article/Supplementary material, further inquiries can be directed to the corresponding authors.
