# Supplementary material for: Artificial intelligence-assisted optimization of extraction process, characterization, and functional analysis of globulin from safflower seed meal
Source: Front Nutr. 2025 Nov 17;12:1708593. doi: 10.3389/fnut.2025.1708593 (PMC12667440; doi:10.3389/fnut.2025.1708593)
Supplement: Supplementary file 1 [file Table_1.docx]

**Supplementary Material**

**Artificial intelligence-assisted optimization of extraction process, characterization, and functional analysis of globulin from safflower seed meal**

Keer Xiao ^1†^, Qiaoyu Wang ^1†^, Xinyu Meng ^1^, Ziteng Zhao ^1^, Mukaddas Sai ^1^, Lili Guan ^1^, Qiuyu Lu ^1^, Lingyu Gao ^1^, Jing Yang ^1,2*^, Linna Du ^1*^

^1^Engineering Research Center of the Chinese Ministry of Education for Bioreactor and Pharmaceutical Development, College of Life Science, Jilin Agricultural University, No. 2888, Xincheng Street, Changchun 130118, China

^2^Institute for Safflower Industry Research of Shihezi University/Pharmacy College of Shihezi University/Key Laboratory of Xinjiang Phytomedicine Resource and Utilization, Ministry of Education, Shihezi, 832002, China

*** Correspondence: Corresponding** Author
Linna Du, Ph.D

Engineering Research Center of the Chinese Ministry of Education for Bioreactor and Pharmaceutical Development, College of Life Science, Jilin Agricultural University, Changchun, China

Tel: +86-431-84533421

Email: dulinna0918@163.com

Jing Yang, Ph.D

Engineering Research Center of the Chinese Ministry of Education for Bioreactor and Pharmaceutical Development, College of Life Science, Jilin Agricultural University, Changchun, China

Institute for Safflower Industry Research of Shihezi University/Pharmacy College of Shihezi University/Key Laboratory of Xinjiang Phytomedicine Resource and Utilization, Ministry of Education, Shihezi, China

Tel: +86-431-84533421

Email: jingy@jlau.edu.cn

**Table S1** Comparison between ANN and RSM approaches.

| **Parameters** | **Extraction yield of globulin** | |
| --- | --- | --- |
|  | **RSM** | **ANN** |
| *R*^2^ | 0.93 | 0.90 |
| MSE | 0.07 | 0.08 |
| RMSE | 0.27 | 0.29 |
| ARE | 4.17 | 5.57 |
| AARD (%) | 4.17 | 4.71 |
